# Feasibility, credence, and usefulness of out-of-office cuffless blood pressure monitoring using smartwatch: a population survey

**DOI:** 10.1186/s40885-023-00242-9

**Published:** 2023-06-01

**Authors:** Yongjun Jang, Jong-Mo Seo, Sang-Hyun Ihm, Hae Young Lee, on behalf of the Korean Society of Hypertension

**Affiliations:** 1 Seoul National University College of Medicine, Seoul, South Korea; 2 Department of Electrical and Computer Engineering, College of Engineering, Seoul National University, Seoul, South Korea; 3 Division of Cardiology, Department of Internal Medicine, Bucheon St. Mary’s Hospital, College of Medicine, The Catholic University of Korea, 101 Daehak-Ro, Jongno-Gu, Seoul, 03080 South Korea; 4 Department of Internal Medicine, Seoul National University College of Medicine, Seoul, South Korea; 5 Department of Internal Medicine, Seoul National University Hospital, Seoul, South Korea

**Keywords:** Hypertension, Blood pressure monitoring, Smartwatch, Cuffless blood pressure monitoring, Survey

## Abstract

**Background:**

Cuffless blood pressure (BP) measurement, enabled by recent advances in wearable devices, allows for BP monitoring in daily life. This study aims to evaluate the feasibility, cresdence, and usefulness of cuffless BP monitoring through a population survey.

**Methods:**

During the "Daily BP Measurement with Your Galaxy Watch" campaign held by the Korean Society of Hypertension, participants were asked to share their experiences with cuffless BP measurement using a smartwatch application through an online survey. The questionnaire included questions about age, underlying medical conditions, smartwatch utilization, experience with BP calibration, the reliability of BP values measured by a smartwatch, and willingness to use the BP monitoring function in the future.

**Results:**

A total of 1071 participants responded to the survey. The largest age group (decile) was 50**-**59 years old (33.3%), followed by 40**-**49 years old (29.9%). Although nearly half of the participants (47.5%) had no chronic diseases, 40.1% reported having hypertension. BP monitoring was the most frequently utilized smartwatch function (95.8%), followed by heart rate measurement (87.1%). 31.8% of participants reported that BP values measured by the smartphone application were "very accurate and helpful," while 63.5% rated them as "slightly lower (44.4%)" or "higher (19.1%)" compared to the standard home BP monitoring device. 93% of the participants reported utilizing the BP monitoring function at least once a week. Regarding the BP calibration process, most participants (93.9%) calibrated the BP measurement application themselves, and 50.8% rated the difficulty level as "very easy."

**Conclusion:**

Cuffless BP measurement using a smartwatch application was feasible in the general population, including the self-calibration process. However, the satisfaction level in terms of accuracy is still modest, indicating a need for further development.

## Background

Hypertension is a significant and modifiable risk factor of cardiovascular disease (CVD) [1, 2]. However, it is often poorly recognized by the general public, despite affecting over one-third of the global adult population. The May Measurement Month 2019 Korea campaign, which enrolled over 9,000 participants in an opportunistic screening program, found that approximately one-third had uncontrolled blood pressure (BP). However, over 20% of participants had not measured their BP in the last 12 months [3]. Younger individuals are even more ignorant of their BP, although the prevalence of hypertension is already substantial from the third decade of life [3, 4]. According to the Korean National Health and Nutrition Examination Survey (KNHANES-VII), although the prevalence of hypertension in South Korea is 28.6% in adult males and 16.8% in adult females, the awareness rate does not exceed 70% [5, 6]. Under such circumstances, regular BP follow-up is challenging, and serious BP-related medical conditions may occur before hypertension diagnosis [7, 8].

Regular BP measurement is, however, important among the established hypertensive population. Since recent clinical trial data invariably showed the benefits of intensive BP lowering [9, 10], accurate BP measurement becomes even more crucial. It is a well-known issue that there is a considerable portion of patients with a white-coat or masked effect [11–13]. Moreover, since BP has large variability throughout every day and every season, regular and frequent BP monitoring is closely related to CVD risk and mortality [8, 14].

Recent advances in smartphones and wearable device technologies evolves their role as a promising tool for long-term out-of-office BP monitoring [7, 15]. Smartphones are widely adopted in daily life, with smartphone users exceeding 2.5 billion in 2019, according to a Pew Research Centre survey conducted among 30,133 people in 27 countries, with South Korea having the highest rate reaching 95% [16]. Recently, the Korean Society of Hypertension (KSH) and the European Society of Hypertension Working Group on Blood Pressure Monitoring and Cardiovascular Variability have published position papers, respectively [7, 15]. These papers indicate the potential role of cuffless BP monitoring using a smartwatch application (App). The position papers commonly indicate that cuffless BP monitoring enables patients with hypertension and healthy individuals to measure their own BP in their daily life.

An example of these mobile healthcare technologies is the Samsung Health Monitor App of the Samsung Galaxy Watch. This application measures BP on a smartwatch by pulse wave analysis (PWA) method with photoplethysmography (PPG) technology and was approved as a Software as a Medical Device (SaMD) by the Ministry of Food and Drug Safety (MFDS) of South Korea in April 2020. In addition, this application met the Association for the Advancement of Medical Instrumentation/European Society of Hypertension/Internal Organization for Standardization (AAMI/ESH/ISO) protocol, which was developed for universal standardization of BP measuring devices by a collaboration of AAMI, ESH, and ISO, and received a CE-marking in December 2020 [17–19].

However, there are few reports regarding user experiences and perceptions of cuffless BP measurement using a smartwatch App. According to a population-based study, there was a statistically significant difference between wrist BP self-measurement at home and reference BP in the general population, even with appropriate training [20]. Additionally, as the Samsung Galaxy Watch uses the PWA method, it is necessary to calibrate the App using a standard BP monitoring device. This process might add further difficulties beyond the measurement process. Therefore, to be widely adopted in the general population and real clinical settings, the measurement and calibration processes should be easy enough for people unfamiliar with those mobile devices to utilize.

In this regard, we conducted a user survey about their experiences and perceptions of cuffless BP monitoring using a smartwatch. This study aims to evaluate the feasibility, credence, and usefulness of cuffless BP monitoring using a smartwatch App in real-world setting.

## Methods

### Recruitment

The event "Daily BP measurement with your Galaxy Watch" was organized by the KSH from May 25 to June 8, 2022, to raise awareness of the public about the usefulness of cuffless BP monitoring using a smartwatch. Interested participants voluntarily signed up on the KSH website (http://www.koreanhypertension.co.kr), and only adults (22 years or older) were eligible to participate since the Samsung Health Monitor App is only available for adults. Before participating in the event, the participants were asked to sign an informed consent form available on the KSH website. The research protocol for this event was approved by the institutional review board of Seoul National University Hospital (H-2207–026-1337).

To promote the event, the KSH used various social media platforms such as Naver blog, Facebook, Instagram, and Kakao Talk channel. Participants were incentivized to participate through giveaways, which were provided by lottery among those who correctly performed the BP calibration, BP measurement, and transmitting procedure as per the provided instructions during the event period. The manufacturer of the device was not involved in the study, including study design, conduction, and data interpretation.

### Participants’ survey

During the event, participants of the event were requested to share their BP measurement experience using a smartwatch App through online survey. The questionnaires are divided into three areas. First part was the questions about participants` characteristics, including age, underlying medical condition, and the length of the smartwatch use. Second part was the question about the status of smartwatch utilization, including the question about health-related functions of the smartwatch, frequency of exercise with wearing the smartwatch, users` perception about accuracy and reliability of BP monitoring function, and willingness to use the BP monitoring function in the future. The third area was the question about the BP calibration process, including feasibility and the way of the calibration process. Questions included in the questionnaire are translated and listed in Table 1. There were no additional exclusion criteria. All responses sent by event participants were included.

**Table 1:** Survey questionnaires

No	Questions
1	What is your age?
2	Do you have any underlying medical conditions? (Select all)
3	How long do you use your smartwatch?
4	Please select all smartwatches that you have used
5	How many times a week do you exercise with your smartwatch?
6	Among the health monitoring applications of the Galaxy Watch, which do you utilize?
7	Which arm did you use to calibrate the Galaxy Watch blood pressure (BP) measurement application?
8	Who performs the BP calibration process of the Galaxy Watch BP measurement application?
9	How much do you estimate the difficulty level of the calibration process of the Galaxy Watch BP measurement application? (Out of 10 points)
10	How do you compare the accuracy of the Galaxy Watch BP measurement application with a standard home BP measurement device?
11	How often will you use the BP measurement application after the event?

### Data collection and statistical analysis

During the campaign, participants were asked to calibrate the smartwatch using an automated upper-arm cuff-based BP monitor approved by the Ministry of Food and Drug Safety. Step-by-step instruction for BP calibration was as follows according to the position statement of the KSH [15]: (i) avoid alcohol, caffeine, and nicotine intake, exercise, and bathing 30 min before correction; (ii) sit comfortably with your back in a chair; (iii) wear your smartwatch on the wrist, without overtightening; (iv) position the cuff around the middle of the upper arm on the same side wearing smartwatch; (v) tap ‘start calibration’ button on the smartphone connected to the smartwatch; (vi) take a reference BP value by the cuff-based BP monitoring device; (vii) put the BP value appeared on the cuff-based BP monitoring device in the smartwatch application; (viii) repeat these for two more times. After the first calibration, participants were instructed to measure their BP twice daily, morning and evening. After a total of 14 measurements over a week, they were instructed to recalibrate their smartwatch. After the second calibration, they were asked to measure their BP twice a day again for one more week, just as they had done before. After every measurement was made, participants were instructed to extract their BP reading data from the Samsung Health Monitor App and send their BP measurement results to the Kakao Talk channel or e-mail address of the KSH in the form of a PDF file. It is recommended to select the last three months from last week, last month, last three months, and last year as the extraction period option so that data for the entire period of the event can be extracted without loss. All personally identifiable information transferred was used for research and analysis in a form in which individuals cannot be identified.

No specific statistical method or statistics program was used to analyze the survey results, and survey data were analyzed mainly with percentage (%) values.

## Results

### Participants’ characteristics

**Fig. 1:**
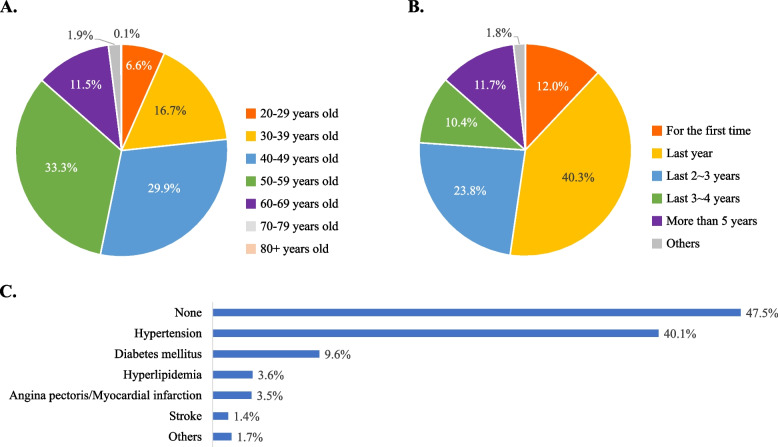
Baseline characteristics of participants. **A** Age distribution divided into ten years. **B** Duration of smartwatch usage. **C** Combined medical conditions (Multiple choice)

A total of 13,539 people applied for the event "Daily BP measurement with your Galaxy Watch." Among the participants who applied and received the questionnaire, 1,071 people responded to the survey. The largest age group was 50–59 years old (33.3%), followed by 40–49 years old (29.9%) and 30–39 years old (16.7%). The population group of 20–29 years old accounted for 6.6% of the total, and only 2.0% were over 70 (Fig. 1A). Regarding the length of smartwatch usage, 86.2% of participants had been using a smartwatch for more than a year, with 45.9% using it for more than 2–3 years, 22.1% for more than 3–4 years, and 11.7% for more than five years. Only 12% of participants were first-time smartwatch users at this event (Fig. 1B). Regarding underlying medical conditions, nearly half (47.5%) of the participants responded that they had no history of chronic disease, while 40.1% reported having hypertension, followed by diabetes mellitus (9.6%), hyperlipidemia (3.6%), angina pectoris/myocardial infarction (3.5%), stroke (1.4%), and other conditions (1.7%) (Fig. 1C). Detailed information about participants’ age distribution, duration of smartwatch usage, and underlying medical conditions is shown in Fig. 1.

### Status of the smartwatch utilization

**Fig. 2:**
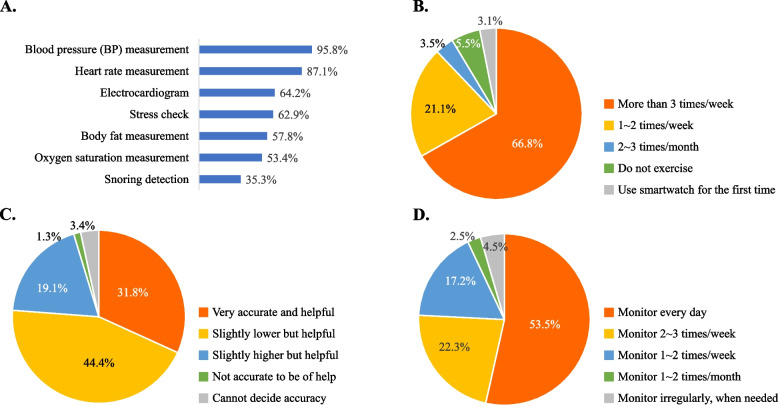
Status of smartwatch utilization, including BP monitoring function. **A** Utilization of health-related applications (Multiple choice). **B** Frequency of exercise per week using smartwatch. **C** Perceived accuracy and usefulness of BP monitoring application. D Willingness to further use the BP monitoring application after the survey

Among the health-related apps built into the smartwatch, BP monitoring (95.8%) was the most commonly utilized app, followed by heart rate measurement (87.1%), electrocardiogram (64.2%), stress evaluation (62.9%), body fat examination (57.8%), oxygen saturation measurement (53.4%), and snoring detection (35.3%) (see Fig. 2A). With regards to the frequency of exercise using a smartwatch, 66.8% of users exercised while wearing a smartwatch at least three times a week. 21.1% exercised 1**-**2 times a week with a smartwatch, while 3.5% exercised 2**-**3 times a month with a smartwatch. 5.5% had never exercised while wearing a smartwatch (see Fig. 2B). The most frequently used smartwatch model was the Galaxy Watch 4 (44.7%), followed by the Samsung Galaxy Watch 3 (26.7%), Samsung Galaxy Watch Active 2 (19.9%), Samsung Galaxy Watch 4 Classic (19%), Samsung Galaxy Watch (17%), and Samsung Galaxy Watch 2 (11.3%). The Apple Watch 1–6, Galaxy Fit, and Mi Band were used by 1–2% of participants for each model.

In terms of the usefulness and reliability of the BP monitoring app on smartwatches, 31.8% of participants answered that the smartwatch’s BP monitoring was ‘very accurate and helpful’. 44.4% rated it as ‘displaying slightly lower BP levels than their conventional counterpart but still helpful’, and 19.1% think that it ‘displays slightly higher BP levels than their conventional counterpart but still helpful’. Only 1.3% said it was ‘not accurate to be of help to’ (see Fig. 2C). When asked about their intention to use the BP monitoring function in the future, 93% of participants said they would use it at least once a week regularly in the future, with 53.5% saying they would use it daily (see Fig. 2D). Detailed information about the status of smartwatch utilization is shown in Fig. 2.

### Experience with BP calibration using a smartwatch

**Fig. 3:**
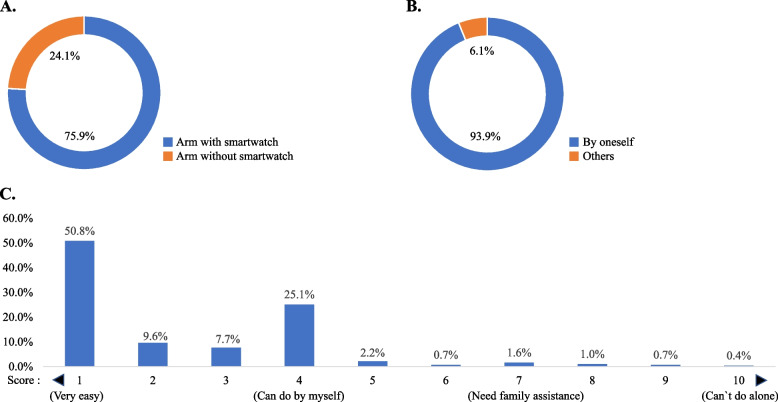
Feasibility of BP calibration process of smartwatch BP monitoring application. **A** Arm used in measurement by standard home BP monitoring device. **B** Assistance of the calibration process. **C** Self-estimation of difficulty in BP calibration (10-point scale). Score 1 means ‘very easy’, Score 4 means ‘can do by myself’, Score 7 means ‘need family assistance’, and Score 10 means ‘can`t do alone’

Regarding the calibration process of a smartwatch for BP measurement, 75.9% of participants used the arm wearing the smartwatch for calibration, while 24.1% used the arm not wearing the smartwatch (Fig. 3A). The reason for including this question is that the BP measurement manual of the Samsung Galaxy Watch and the KSH’s recommendations for BP calibration differ. The manual for the Samsung Galaxy Watch recommends wearing a cuff-type BP monitoring device on the arm not wearing the smartwatch during BP calibration, but the KSH recommends wearing it on the arm wearing the smartwatch.

Most participants (93.9%) were able to calibrate their BP by themselves without needing help from others (Fig. 3B). Regarding the difficulty of the calibration procedure with a cuff-based BP monitoring device, 93.2% of responders rated the process as easy enough for them to do by themselves without assistance. Among them, 50.8% rated the difficulty of the BP calibration process as ‘very easy’. Only 3.7% said they needed family assistance to perform BP calibration, and only 0.4% said they could never calibrate their BP by themselves (Fig. 3C). Detailed information about the feasibility and users’ perception of BP calibration using a smartwatch BP monitoring application is shown in Fig. 3.

## Discussion

The main findings of this study can be summarized as follows: Smartwatches were widely utilized in the middle-aged group. Although the use of BP measurement using a smartwatch app in the hypertensive population is not recommended in the position papers from the academic societies as well as the producing company, about 40% of the users had hypertension. More than 50% of the participants found the BP monitoring function of a smartwatch quite useful and were willing to utilize it daily. However, only one-third of the participants responded that the accuracy of the smartwatch’s BP monitoring app was comparable to the standard home BP measurement device. Most users were able to perform BP calibration on their own without assistance. To the best of our knowledge, this study is the first to investigate the real users’ perception of the BP measuring app of a smartwatch.

Noticeably, middle-aged populations widely utilize smartwatches, contrary to the common thought that smartwatches are mainly used in the younger generation. Additionally, approximately 40% of smartwatch users had hypertension as an underlying medical condition. Until now, the utilization of BP monitoring apps in smartwatches as a medical tool is not recommended in patients with underlying medical conditions like diabetes mellitus, cardiomyopathy, end-stage renal disease (ESRD), and high systolic BP ≥ 160 mmHg or low diastolic BP ≤ 60 mmHg [15]. However, it is remarkable that smartwatches are already being utilized by those with underlying diseases, including hypertensive patients. Therefore, further studies investigating the accuracy of BP measurement in patients with underlying diseases, including hypertension, are warranted.

The results of this survey suggest that BP monitoring using a smartwatch has an advantage in terms of user compliance, and the high willingness to utilize the BP monitoring App supports the possibility of using the smartwatch as a medical device for long-term out-of-hospital BP monitoring. However, the perceived accuracy level of the BP measuring function by the users is limited, with 63.5% of the participants rating the accuracy of BP values measured by a smartwatch as less accurate than conventional BP measuring devices. Technical development is needed to meet the high standard of the current medical standard, especially in the hypertensive range of BP.

Regarding the BP calibration procedure of the smartwatch using PPG technology, it was feasible for most smartwatch users to calibrate the BP of the Samsung Health Monitor application with a ‘conventional’ home BP measurement device for themselves without assistance from others. We asked whether users used the same arm or the contralateral arm for calibration because the Samsung company and the position paper from the KSH recommended different arms. The manual of the Samsung Galaxy Watch recommends positioning the cuff on the user’s arm not wearing a smartwatch, while the KSH recommends putting the cuff on the arm wearing a smartwatch. The method recommended by the company has the benefit of simultaneous measurement between the BP measurement App and the standard home BP measurement device, thus eliminating time-related bias. In contrast, the KSH method pays attention to the BP difference between the two arms. According to epidemiological studies, the mean inter-arm BP difference was 3.3 mmHg for SBP and 2.0 mmHg for DBP [21]. This inter-arm difference can cause an intrinsic ~ 3 mmHg error that cannot be corrected when using the arm not wearing a smartwatch. Additionally, using the contralateral arm for calibration might not provide the benefit of simultaneous measurement in reality. If there is someone to help the user, like research nurses in a clinical trial, the user can measure the BP in both arms and check the difference in SBP between the arms. However, in real-world settings, users cannot calibrate BP and use cuff-based BP monitoring devices simultaneously without help. Therefore, since there is no advantage to using the other arm with this inter-arm BP difference, the KSH position paper recommends using the arm wearing a smartwatch [15], and the response in this survey also support the KSH method.

This study has some possible limitations, such as the prize giveaway during the event which could have influenced participants to respond in a more positive direction, and a potential selection bias as the survey was conducted among those who voluntarily participated in the event. Additionally, the high cost of a smartwatch may have biased the age composition of participants.

## Conclusions

Cuffless BP monitoring using a smartwatch App is feasible in the general population, including the self-calibration process. However, the accuracy level needs further development to meet the current medical standard.

## Data Availability

The data supporting this study’s findings are available from the corresponding author upon reasonable request.
